# Differential expression of cancer‐related genes supports prediction of poor response to first‐line treatments in T‐ALL pediatric patients with high minimal residual disease

**DOI:** 10.1002/1878-0261.70234

**Published:** 2026-04-09

**Authors:** Antonio Lahera, Laura Vela‐Martín, Pablo Fernández‐Navarro, José Luis López‐Lorenzo, Javier Cornago, Pilar Llamas, Eduardo Pérez‐Gómez, José Luis Marín‐Rubio, Manuel Fresno, Javier Santos, José Fernández‐Piqueras, María Villa‐Morales

**Affiliations:** ^1^ Department of Molecular Biology Universidad Autónoma de Madrid Spain; ^2^ Centro de Biologia Molecular Severo Ochoa (CBM) CSIC ‐ Universidad Autonoma de Madrid Spain; ^3^ Department of Biology Universidad Autónoma de Madrid Spain; ^4^ Area of Genetics and Genomics IIS Fundación Jiménez Díaz Madrid Spain; ^5^ Unit of Cancer and Environmental Epidemiology, Centro Nacional de Epidemiología Instituto de Salud Carlos III Madrid Spain; ^6^ Networking Biomedical Research Centre of Epidemiology and Public Health (CIBERESP) Madrid Spain; ^7^ Division of Hematology and Hemotherapy IIS Fundación Jiménez Díaz Madrid Spain; ^8^ Departamento de Bioquímica y Biología Molecular Universidad Complutense de Madrid Spain; ^9^ Instituto de Investigación Hospital 12 de Octubre Madrid Spain; ^10^ Human Oncology & Pathogenesis Program, Sloan Kettering Institute Memorial Sloan Kettering Cancer Center New York New York USA; ^11^ Instituto de Investigación Sanitaria Hospital Universitario de La Princesa Madrid Spain; ^12^ Institute for Molecular Biology‐IUBM (Universidad Autónoma de Madrid) Madrid Spain

**Keywords:** early biomarker, minimal residual disease (MRD), response to therapy, T‐cell acute lymphoblastic leukemia (T‐ALL)

## Abstract

T‐cell acute lymphoblastic leukemia (T‐ALL) originates from the malignant transformation of immature lymphoblasts committed to the T‐cell lineage. Relapsed or refractory T‐ALL patients show a dismal outcome with limited therapeutic options and cure rates below 10%. Accurate risk stratification is essential for optimizing first‐line treatments and maximizing initial complete response rates. Minimal residual disease (MRD) assessment is the most relevant clinical parameter in T‐ALL and a direct measure of treatment response, but it requires an initial treatment course that reduces the time for decision‐making. Consequently, there is an urgent need to identify novel biomarkers that can predict the response to first‐line treatments at diagnosis. By integrating clinical and transcriptomic data from diagnostic T‐ALL samples, we found that high MRD patients show a specific transcriptional profile. Moreover, we identified a transcriptional signature characterized by the differential expression of *HSH2D*, *LAT2*, *BCL2*, *MAST4*, *METRN*, and *PITPNM2* genes that is tightly associated with an increased MRD, which could improve the prediction of poor treatment response in T‐ALL patients, especially during early treatment phases.

## Graphical abstract

In the present work, we have identified a transcriptional signature based on the differential expression of six genes (*BCL2*&*MAST4*, *HSH2D*&*LAT2*, *METRN*&*PITPNM2)* that would facilitate the early detection of T‐cell acute lymphoblastic leukemia (T‐ALL) patients prone to a poor treatment response and could be implemented at diagnosis, along with other risk factors that are normally considered for risk assessment in T‐ALL.

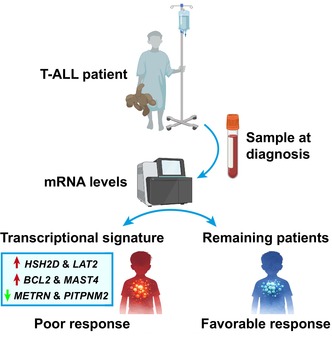

**Abbreviations**
FDR: false discovery rate
MCA: multiple correspondence analysis
MRD: minimal residual disease
NES: normalized enrichment score
T‐ALL: T‐cell acute lymphoblastic leukemia
TARGET: Therapeutically Applicable Research to Generate Effective Treatments

## 1 Introduction

Acute lymphoblastic leukemia (ALL) is an aggressive hematological malignancy that represents the most common childhood cancer and a significant cause of mortality in adults. ALL arises from the malignant transformation of lymphoid precursors and is classified according to the cell of origin as B‐lineage acute lymphoblastic leukemia (B‐ALL) or T‐cell acute lymphoblastic leukemia (T‐ALL), with marked differences in their incidence and mutational spectrum. The incidence of B‐ALL is higher and accounts for about 80% of ALL, while T‐ALL only accounts for about 20% of cases [1]. Moreover, the mutational spectrum is more heterogeneous in T‐ALL and, accordingly, T‐ALL patients have been recently classified into 15 different genomic subtypes [2].

Consequently, several personalized therapies, such as tyrosine kinase inhibitors, and immunotherapies, including anti‐CD19/CD22 CAR T cells, have been successfully developed for B‐ALL. In contrast, there are no targeted treatments approved for newly diagnosed T‐ALL patients and the last pharmacological inhibitor specifically implemented for refractory or relapsed cases was nelarabine in 2005, so the therapeutic options remain limited [3]. As a result, those T‐ALL patients who do not respond to first‐line treatments or experience a relapse have dismal prognosis, with cure rates below 10%. Therefore, current efforts are focused on optimizing first‐line treatments and maximizing initial complete response rates. In this scenario, molecular markers capable of predicting the initial response to first‐line treatments are essential for appropriate risk stratification, so high‐risk patients may benefit from more intensive chemotherapy regimens and hematopoietic stem cell transplantation, while low‐risk patients may benefit from less intensive treatment approaches, which are characterized by reduced toxicity and fewer side effects [4]. Similar to personalized therapies, molecular markers are less clear in T‐ALL than in B‐ALL. At present, the most relevant clinical parameter for T‐ALL is the assessment of minimal residual disease (MRD), which is generally defined as the proportion of leukemic blasts relative to the total number of nucleated cells [3]. MRD represents a direct measure of treatment response, as low MRD indicates favorable response, while high MRD reflects poor response [5]. Thus, MRD assessment after induction chemotherapy provides valuable information about initial tumor aggressiveness and first‐line treatment response [6]. However, MRD cannot be assessed from the beginning but inevitably requires an initial treatment course, limiting its utility for early decision‐making and potentially compromising optimal clinical management [7].

As a result, a promising approach to improve the clinical management of T‐ALL patients would be the identification of novel molecular markers that are tightly associated with a poor initial response to first‐line treatments and thus, with high MRD values after induction chemotherapy, but that can also be evaluated at diagnosis and first biopsy analysis without the need for an initial treatment course [7]. Since the molecular bases underlying high MRD values after induction chemotherapy remain largely unexplored, we hypothesized that transcriptomic profiling of diagnostic T‐ALL samples stratified by MRD status could reveal genes that are differentially expressed in high MRD patients and may become novel biomarkers for improved risk stratification. Additionally, such genes would be shared by a substantial fraction of high MRD patients, especially since induction chemotherapy regimens are relatively uniform and mostly involve glucocorticoids, anthracyclines and vincristine [4, 8].

## 2 Materials and methods

### 2.1 Human samples

To investigate the molecular basis underlying high MRD in T‐ALL patients, we analyzed data from 265 T‐ALL patients enrolled in the Therapeutically Applicable Research to Generate Effective Treatments (TARGET) initiative. The results published here are in whole or part based upon data generated by the TARGET initiative (https://www.cancer.gov/ccg/research/genome‐sequencing/target), phs000218, managed by the National Cancer Institute. Data used for this analysis are accessible in the database of Genotypes and Phenotypes (dbGaP, https://www.ncbi.nlm.nih.gov/projects/gap/cgi‐bin/study.cgi?study_id=phs000464.v23.p8), accession number phs000218.v26.p8 and substudy specific accession number phs000464.v23.p8 (TARGET Acute Lymphoblastic Leukemia (ALL) Expansion Phase 2).

The results were further validated in 1070 additional T‐ALL patients from Gabriella Miller Kids First Pediatric Research Program projects. The results analyzed and shown here are based in whole or in part upon data generated by Gabriella Miller Kids First Pediatric Research Program projects and by The National Cancer Institute's Childhood Cancer Data Initiative (CCDI) supplemental funding to share data generated in this project with the research community under grant No. 3P30CA008748‐54S3. Data used for this analysis are accessible in the dbGaP (https://www.ncbi.nlm.nih.gov/projects/gap/cgi‐bin/study.cgi?study_id=phs002276.v3.p1), specific accession number phs002276.v3.p1 (Gabriella Miller Kids First Pediatric Research Program in Pediatric T‐Cell Acute Lymphoblastic Leukemia).

All patients reported informed consent or assent before study entry according to the Declaration of Helsinki and received a homogeneous first‐line treatment according to the Children's Oncology Group (COG) clinical trial AALL0434. Eligible cases included patients aged 1–31 years who were diagnosed with T‐ALL between January 2007 and July 2014 and had not received any prior treatment, except for corticosteroids.

Patients from the Gabriella Miller Kids First Pediatric Research Program were classified according to the 15 genomic subtypes established by Pölönen *et al*. using the information provided in their manuscript [2].

### 2.2 Statistical analysis

The Shapiro–Wilk and Anderson–Darling tests were used to check for normality of data groups. Differential gene expression analysis was performed in R using the DESeq2 Bioconductor package for differential expression testing, which models RNA‐seq count data using a negative binomial distribution. Differences between high and low MRD cases were assessed using the Wald statistic, with *P*‐values adjusted for multiple testing using the Benjamini–Hochberg procedure. Genes with an absolute log_2_ fold change greater than 1 and an adjusted *P*‐value below 0.05 were initially selected. These genes were subsequently ranked according to their Wald statistics and following the ranking principle underlying Gene Set Enrichment Analysis (GSEA). Differences between independent samples were analyzed using the non‐parametric Mann–Whitney test for those variables not adjusted to normality. Significant differences regarding association analysis were assessed through contingency analysis using chi‐square, Fisher's exact test or binomial exact test. *P*‐values were adjusted using the Benjamini–Hochberg multiplicity test. Proportions are reported with 95% confidence intervals (CIs) calculated using the Wilson method. Kaplan–Meier curves for event‐free survival (EFS) analysis were plotted using GraphPad Prism. The log‐rank (Mantel–Cox) test was used to determine the *P*‐value and the Mantel–Haenszel test was used to determine the hazard ratio. Cumulative incidence of relapse (CIR) analysis was performed using Gray test using the “cmprsk” (v2.2–12) R package.

To assess the relationship between MRD after induction chemotherapy and differentially expressed genes at diagnosis, we employed multiple correspondence analysis (MCA). For each of the selected genes, patients were divided into quartiles according to their mRNA expression levels and then classified as Q1 (lowest expression); Q2; Q3; or Q4 (highest expression). The number of components was set at 2, as they accounted for more than 60% of the total variance, in agreement with recommendations previously reported in the literature [9]. *K*‐means was applied for sample clustering and the number of clusters (*k*) was also set at 2 according to both elbow method and silhouette analysis. The transcriptional signature was generated from the subset of 25 genes on the basis of three main premises: (1) to maximize the number of patients with high MRD included in the signature, (2) to minimize the number of patients with low MRD included in the signature, and (3) to ensure that patients included in the transcriptional signature were identified by at least two different genes. All possible gene combinations were systematically evaluated using Python and R in accordance with these three criteria. The gene subset that achieved the highest score (defined as the greatest number of high MRD patients and the lowest number of low MRD patients, while ensuring that each patient was identified by at least two genes) was selected for subsequent analyses.

Statistical analyses were performed using graphpad prism RRID:SCR_002798 version 8, R 4.0.3 and Python 3.13.2. Statistical significance was set at *P* < 0.05 for all statistical analyses.

## 3 Results

**Fig. 1:**
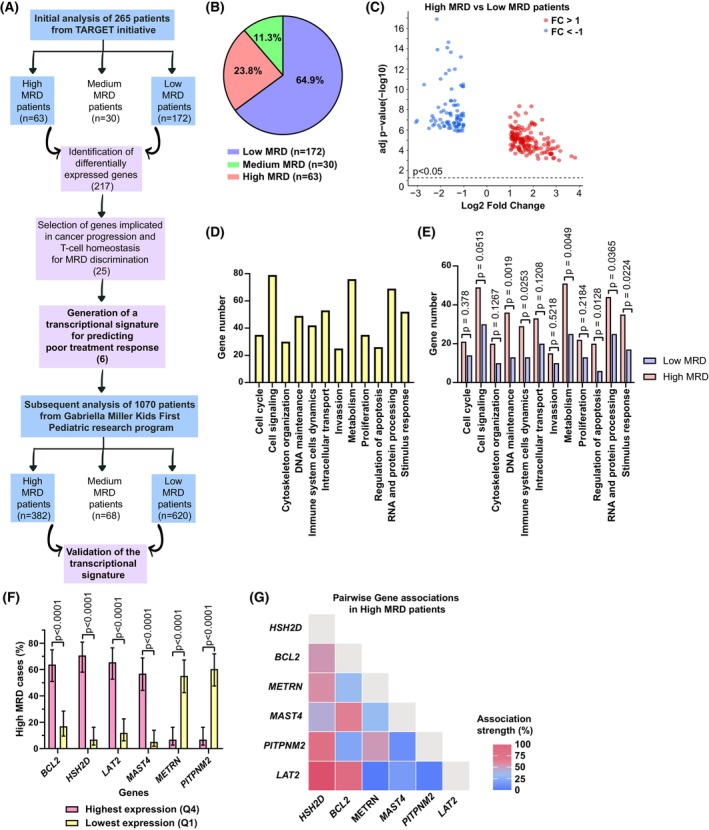
Study of the molecular basis underlying high minimal residual disease (MRD) levels in T‐cell acute lymphoblastic leukemia (T‐ALL). (A) Schematic representation of the flowchart followed to identify the genes that constitute the transcriptional signature which predicts poor treatment response. (B) Classification of T‐ALL patients from Therapeutically Applicable Research to Generate Effective Treatments (TARGET) initiative according to their MRD levels after induction chemotherapy. MRD low: < 0.01%; MRD medium: 0.01–0.1%; MRD high: > 0.1%. (C) Volcano plot showing those differentially expressed genes (log_2_FC > |1| and *P* < 0.05) that exhibited the most consistent and robust expression differences according to their Wald statistic derived from the DESeq2 analysis and following the ranking principle underlying Gene Set Enrichment Analysis (GSEA). (D) Classification of the differentially expressed genes between high and low MRD patients according to their association with several biological processes that are relevant for T‐cell homeostasis. The number of genes involved in each cellular process is represented. (E) To determine whether any of the biological processes relevant to T‐cell homeostasis were potentially associated with a specific MRD status, those genes shown in Fig. 1D (differentially expressed genes involved in the selected processes) were further categorized according to their overexpression in high or low MRD patients. Subsequently, the number of overexpressed genes within each biological process was compared between the two MRD subgroups using the chi‐square test and statistical significance was set at *P* < 0.05. (F) Percentage of high MRD patients with upregulation (Q4) or downregulation (Q1) of *BCL2*, *HSH2D*, *LAT2*, *MAST4*, *METRN* and *PITPNM2* genes. For each of the selected genes, patients were divided into quartiles according to their mRNA expression levels and the proportion of high MRD patients was compared between cases with high (Q4) and low (Q1) expression. The data are represented as percentages with 95% confidence intervals (Wilson method). Data were analyzed using Fisher's exact test and statistical significance was set at *P* < 0.05 for all comparisons. (G) Association analysis between high MRD patients with *BCL2*, *HSH2D*, *LAT2*, *MAST4*, *METRN* or *PITPNM2* deregulation. According to the results shown in Fig. 1G, we specifically examined upregulation (Q4) for *BCL2*, *HSH2D*, *LAT2* and *MAST4* genes and downregulation (Q1) for *METRN* and *PITPNM2* genes. Combinations were tested using the chi‐square test and corrected by the Benjamini–Hochberg multiplicity test. The association strength was estimated from the normalized chi‐square association values obtained for each gene pair and statistical significance was set at *P* < 0.05.

To determine whether high MRD patients exhibit a specific transcriptional profile, we analyzed paired clinical and molecular data relative to 265 pediatric T‐ALL cases from the TARGET initiative and specifically looked for differentially expressed genes between high and low MRD patients (Table S1 and Fig. 1A,B). *High MRD patients exhibited* a specific transcriptional profile at diagnosis, *defined by* the differential expression of 2057 genes with an adjusted *P*‐value lower than 0.05 and a log_2_ fold change higher than |1| (Fig. S1A). Among them, 217 genes displayed *the most consistent and robust expression differences* when compared with low MRD patients (Fig. 1C). To explore whether such differentially expressed genes act independently or instead converge on specific biological processes, we assessed their involvement in multiple pathways relevant to T‐cell homeostasis (Fig. 1D). High MRD patients showed marked overexpression of genes associated with apoptosis regulation, DNA maintenance, immune system cell dynamics, RNA/protein processing and metabolism, suggesting that their transcriptional profile at diagnosis may influence treatment response (Fig. 1E).

To evaluate the potential of transcriptomic profiling as a tool to discriminate T‐ALL patients by MRD status, we focused on a specific subset of differentially expressed genes that are implicated in cancer and belong to different cellular processes relevant for T‐cell homeostasis (Fig. S1B). Remarkably, this 25‐gene subset did not result in a random distribution but instead followed a specific pattern and divided T‐ALL patients into two distinct clusters (Fig. S1C). We proved that these clusters showed significant differences in spatial distribution (Fig. S1D) and MRD values (Fig. S1E), supporting transcriptomic profiling as a potential approach for discriminating T‐ALL patients by MRD status.

Consequently, a transcriptional signature based on the differential expression of a few genes could become an easy‐to‐implement method for predicting poor treatment response and identifying those T‐ALL patients likely to experience high MRD values after induction chemotherapy without the need to wait for a treatment course. From the previous 25‐gene subset, we systematically evaluated which transcriptional signatures potentially involved the greatest number of high MRD patients and the lowest number of low MRD patients, while ensuring that each patient was identified by at least two genes. Next, we examined the six genes included in the selected transcriptional signature and confirmed that the differential expression of each gene was associated with higher MRD values (Fig. S2A). Moreover, we observed that a greater proportion of high MRD patients expressed the highest mRNA levels of *BCL2*, *HSH2D*, *LAT2*, and *MAST4* genes (Q4) and the lowest mRNA levels of *METRN* and *PITPNM2* (Q1) (Fig. 1F).

**Fig. 2:**
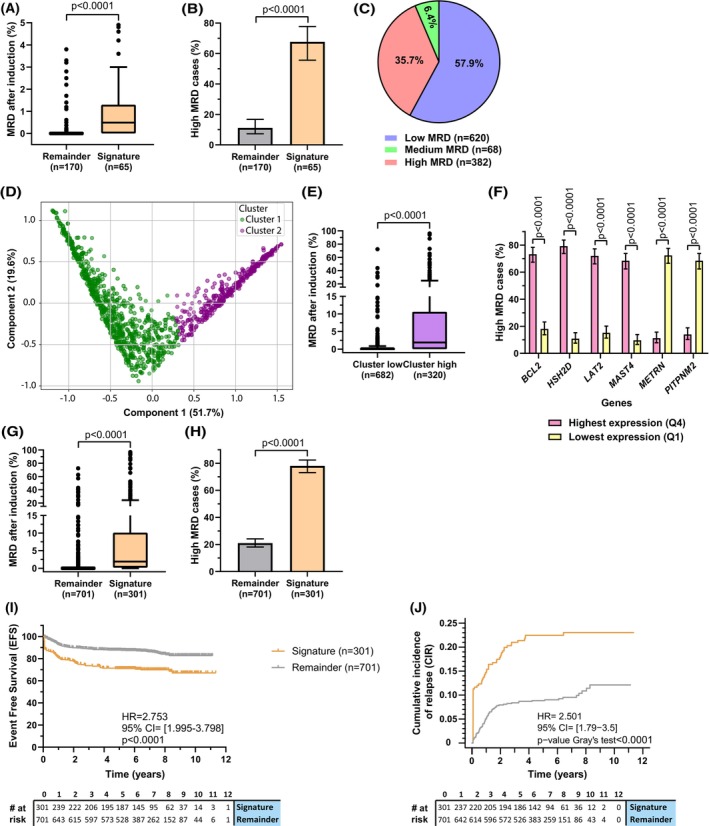
Differential expression of *HSH2D*&*LAT2*/*BCL2*&*MAST4*/*METRN*&*PITPNM2* at diagnosis predicts high minimal residual disease (MRD) levels in T‐cell acute lymphoblastic leukemia (T‐ALL) patients. (A) Boxplot comparing MRD levels after induction chemotherapy between those patients included in the transcriptional signature and the remaining T‐ALL patients from Therapeutically Applicable Research to Generate Effective Treatments (TARGET) initiative. The transcriptional signature involves those cases with simultaneous upregulation (Q4) of either *HSH2D* and *METRN* or *BCL2* and *MAST4* or simultaneous downregulation (Q1) of *METRN* and *PITPNM2*. Only those patients with MRD values classified as either high or low were considered for analysis. Data were analyzed using Mann–Whitney test and statistical significance was set at *P* < 0.05. (B) Probability of experiencing a high MRD after induction chemotherapy for those patients included in the transcriptional signature and compared to the rest of T‐ALL patients from TARGET initiative. The graph shows the percentage of high MRD cases with 95% confidence interval (Wilson method). Data were analyzed using Fisher's exact test and statistical significance was set at *P* < 0.05. (C) Classification of T‐ALL patients from Gabriella Miller Kids First Pediatric Research Program according to their MRD levels after induction chemotherapy. MRD low: < 0.01%; MRD medium: 0.01–0.1%; MRD high: > 0.1%. (D) Multiple correspondence analysis (MCA) for T‐ALL patients according to the differential expression of the gene subset previously specified in Fig. S1B. For each of the selected genes, patients were divided into quartiles according to their mRNA expression levels and subsequently classified as Q1 (lowest expression); Q2; Q3; or Q4 (highest expression). Only those patients with MRD values classified as either high or low were considered for analysis. (E) Boxplot comparing MRD levels after induction chemotherapy between patients from cluster 1 and cluster 2. Data were analyzed using Mann–Whitney test and statistical significance was set at *P* < 0.05. (F) Percentage of high MRD patients with upregulation (Q4) or downregulation (Q1) of *BCL2*, *HSH2D*, *LAT2*, *MAST4*, *METRN* and *PITPNM2* genes. For each of the selected genes, patients were divided into quartiles according to their mRNA expression levels and the proportion of high MRD patients was compared between cases with high (Q4) and low (Q1) expression. The data are represented as percentages with 95% confidence intervals (Wilson method). Data were analyzed using Fisher's exact test and statistical significance was set at *P* < 0.05 for all comparisons. (G) Boxplot comparing MRD levels after induction chemotherapy between those patients included in the transcriptional signature and the remaining T‐ALL patients from Gabriella Miller Kids First Pediatric Research program. The transcriptional signature involves those cases with simultaneous upregulation (Q4) of either *HSH2D* and *METRN* or *BCL2* and *MAST4* or simultaneous downregulation (Q1) of *METRN* and *PITPNM2*. Only those patients with MRD values classified as either high or low were considered for analysis. Data were analyzed using Mann–Whitney test and statistical significance was set at *P* < 0.05. (H) Probability of experiencing a high MRD after induction chemotherapy for those patients included in the transcriptional signature and compared to the rest of T‐ALL patients from Gabriella Miller Kids First Pediatric Research Program. The graph shows the percentage of high MRD cases with 95% confidence interval (Wilson method). Data were analyzed using Fisher's exact test and statistical significance was set at *P* < 0.05. (I) Kaplan–Meier curves comparing event‐free survival (EFS) between patients from Gabriella Miller Kids First Pediatric Research Program included in the transcriptional signature and the remaining patients. The log‐rank (Mantel–Cox) test was used to determine the p‐value and the Mantel–Haenszel test was used to determine the hazard ratio. Statistical significance was set at *P* < 0.05. (J) Cumulative incidence of relapse (CIR) for those patients included in the transcriptional signature and the rest of patients. CIR analyses were performed using Gray test and statistical significance was set at *P* < 0.05.

Association analysis revealed preferential co‐deregulation patterns on the expression levels of candidate genes in high MRD patients. We observed that *LAT2* deregulation preferentially co‐occurred with *HSH2D* and to a lesser extent with *BCL2*. There was a tight association between *BCL2* and *MAST4* upregulation as well as between *METRN* and *PITPNM2* downregulation (Fig. 1G). These results suggested that candidate genes may work in pairs and lead to more refined selection criteria, so we first confirmed the existence of significant associations between the different pairs and then postulated a transcriptional signature based on the simultaneous deregulation of either *HSH2D* and *LAT2* or *BCL2* and *MAST4* or *METRN* and *PITPNM2* (*HSH2D* & *LAT2*/ *BCL2* & *MAST4*/ *METRN* & *PITPNM2*) (Fig. S2B). Patients positive for this transcriptional signature exhibited significantly higher MRD values than the remaining patients and similar to those previously observed for the 25‐gene subset (Fig. 2A). Notably, these patients also had a significantly greater probability of experiencing an elevated MRD (Fig. 2B), further supporting that the transcriptional signature at diagnosis was tightly associated with higher MRD values after induction chemotherapy.

To validate these findings, we analyzed 1070 additional T‐ALL cases from the Gabriella Miller Kids First Pediatric Research Program (Table S2 and Fig. 2C). We first tested that the previous 25‐gene subset again divided T‐ALL patients into two clusters (Fig. 2D) with marked differences in spatial distribution (Fig. S3A) and MRD values (Fig. 2E). Consistently, *BCL2*, *HSH2D*, *LAT2*, *MAST4*, *METRN*, and *PITPNM2* genes, which constitute the transcriptional signature, were also differentially expressed between high and low MRD patients (Fig. S3B). We next confirmed that their differential expression was significantly associated with a greater proportion of high MRD patients (Fig. 2F) and maintained the previously established pairwise correlations (Fig. S3C), reaffirming their suitability for the transcriptional signature. In this additional subset of T‐ALL cases, the transcriptional signature also implied higher MRD values (Fig. 2G) as well as a greater probability of experiencing an elevated MRD (Fig. 2H). Given that a novel classification of T‐ALL patients into 15 genomic subtypes has recently emerged [2], we further assessed the potential of the transcriptional signature through the comparison with the different genomic subtypes (Table S3). The transcriptional signature, along with ETP‐like, KMT2A, LMO2 γδ‐like, and NKX2‐5 genomic subtypes, was significantly associated with a greater probability of experiencing an elevated MRD after induction chemotherapy. The transcriptional signature also demonstrated significant specificity and sensitivity, further supporting its potential as a novel biomarker for predicting poor treatment response. In this line, the transcriptional signature was significantly enriched in patients from the genomic subtypes associated with higher MRD values, whereas most patients belonging to the genomic subtypes associated with lower MRD values tested negative (Fig. S4A,B). Since high MRD levels after induction chemotherapy are often associated with a poor prognosis, we also explored the potential relationship of the transcriptional signature with different clinical adverse events. Patients positive for the transcriptional signature exhibited shorter event‐free survival (EFS) (Fig. 2I) and higher cumulative incidence of relapse (CIR) (Fig. 2J) than the remaining patients, further supporting its association with inferior prognosis.

## 4 Discussion

First‐line treatments are considered an essential stage in the clinical management of T‐ALL, since relapsed or refractory patients have poor prognosis and very low survival rates. In this context, molecular markers capable of predicting the initial response to first‐line treatments could contribute to improve the clinical management of T‐ALL patients [4]. However, such markers are rather scarce in T‐ALL and MRD remains as the most reliable clinical parameter for assessing treatment response after induction chemotherapy [8]. However, MRD assessment requires an initial treatment course, so a promising approach would be the identification of novel molecular markers that are tightly associated with a poor initial response to first‐line treatments and, in consequence, with high MRD values after induction chemotherapy but that can also be assessed at diagnosis and first biopsy analysis without the need for an initial treatment course. Historically, efforts to identify molecular markers in T‐ALL have focused on the immunophenotype and the mutational background. However, this approach drastically reduces the pool of potential candidates and may not be specific enough to reflect the intrinsic complexity of T‐ALL. Immunophenotypic classification comprises only five major subgroups and the number of genes recurrently affected by genetic alterations is around 160 [2, 10]. Consequently, most of the molecular markers proposed for T‐ALL have not been consistent enough to achieve reproducible statistical significance and their clinical application is limited. In this respect, the ETP immunophenotype was initially associated with poor prognosis [11], but subsequent studies have reported contradictory results, probably because the molecular alterations that support leukemogenesis in T‐ALL are very heterogeneous and may not rely exclusively on the immunophenotype [2, 12, 13]. Similarly, mutations in *RAS* or *PTEN* have been associated with adverse outcomes, whereas mutations affecting *NOTCH1* or *FBXW7* have been associated with favorable treatment responses [14]. Nonetheless, the predictive value of these genetic alterations as risk factors remains controversial [2, 15, 16], presumably because: (1) their incidence in T‐ALL is limited (around 14% for *RAS* mutations); (2) different alterations in the same gene may exert variable effects, and thus, their impact on prognosis is not identical; and (3) they coexist with additional alterations that may not be taken into account (as it often happens for *NOTCH1* mutations).

Based on these limitations, we followed an alternative approach and focused on gene expression, since the transcriptional profile of T‐ALL patients with high MRD values after induction chemotherapy had not been explored yet. In this regard, we observed that high MRD patients exhibited a specific transcriptional profile characterized by differential expression of multiple genes involved in different cellular processes that are essential for T‐cell homeostasis. Moreover, we showed that the transcriptional profile was able to discriminate T‐ALL patients by MRD status. Next, we systematically explored multiple gene combinations to identify those transcriptional signatures that potentially involved the greatest number of high‐MRD patients and the lowest number of low MRD patients, while ensuring that each patient was identified by at least two genes. In this respect, we demonstrated that a transcriptional signature based on the differential expression of only six genes predicted poor treatment response and was tightly associated with high MRD values after induction chemotherapy. As with most genetic and epigenetic biomarkers proposed for hematological neoplasms, this transcriptional signature would ideally be further validated in future studies and subsequently considered as an additional risk factor that, in combination with other clinical parameters and molecular markers, would allow a more accurate risk stratification of T‐ALL patients. In this regard, the present transcriptional signature would facilitate the early detection of cases prone to a poor treatment response and could be implemented at diagnosis, along with other risk factors that are normally considered for risk assessment in T‐ALL. Moreover, future research addressing the specific contribution of the genes included in the transcriptional signature to the resistance mechanisms in T‐ALL may reveal whether these genes could also emerge as potential therapeutic targets for the development of novel personalized treatments. In this context, *BCL2* upregulation is a recurrent event in T‐ALL and pharmacological inhibitors targeting BCL2 have already been proposed as a potential therapeutic option for T‐ALL patients [17].

## 5 Conclusions

These results contribute to understand the molecular basis underlying those patients who experience a poor initial response to first‐line treatments and may be useful to improve the clinical management of T‐ALL, especially during the early stages of the disease.

## Conflict of interest

The authors declare no conflict of interest.

## Author contributions

AL, LV‐M, and MV‐M conceived and designed the initial hypothesis and the working plan. AL, LV‐M, and MV‐M performed bioinformatics analysis from patients' data. EP‐G and PF‐N assisted with bioinformatics analysis and data interpretation. JLL‐L, JC, and PL provided clinical advice. JLM‐R, MF, JS, and JF‐P made critical intellectual contributions throughout the project. MF, JS, JF‐P, and MV‐M obtained funding. AL, LV‐M, and MV‐M wrote the original draft, which was reviewed and edited by all authors prior to submission. All authors approved the final version of the manuscript.

## Supporting information


**Table S1.** Therapeutically Applicable Research To Generate Effective Treatments (TARGET) initiative.
**Table S2.** Gabriella Miller Kids First Pediatric Research Program.
**Table S3.** Comparison between the transcriptional signature and the genomic subtypes.
**Fig. S1.** Identification of candidate genes for the transcriptional signature.
**Fig. S2.** Transcriptional signature in T‐cell acute lymphoblastic leukemia (T‐ALL) patients from Therapeutically Applicable Research To Generate Effective Treatments (TARGET) initiative.
**Fig. S3.** Transcriptional signature in T‐cell acute lymphoblastic leukemia (T‐ALL) patients from Gabriella Miller Kids First Pediatric Research Program.
**Fig. S4.** The differential expression of *HSH2D*&*LAT2*/*BCL2*&*MAST4* /METRN&PITPNM2 is associated with high minimal residual disease (MRD) genomic subtypes.

## Data Availability

Data from the Therapeutically Applicable Research to Generate Effective Treatments (TARGET) initiative are managed by the National Cancer Institute and accessible in the database of Genotypes and Phenotypes (dbGaP, https://www.ncbi.nlm.nih.gov/projects/gap/cgi‐bin/study.cgi?study_id=phs000464.v23.p8), accession number phs000218.v26.p8 and substudy specific accession number phs000464.v23.p8 (TARGET Acute Lymphoblastic Leukemia (ALL) Expansion Phase 2). Data from Gabriella Miller Kids First Pediatric Research Program are accessible in the dbGaP (https://www.ncbi.nlm.nih.gov/projects/gap/cgi‐bin/study.cgi?study_id=phs002276.v3.p1), specific accession number phs002276.v3.p1 (Gabriella Miller Kids First Pediatric Research Program in Pediatric T‐Cell Acute Lymphoblastic Leukemia).
