# Supplementary material for: Differential expression of cancer‐related genes supports prediction of poor response to first‐line treatments in T‐ALL pediatric patients with high minimal residual disease
Source: Mol Oncol. 2026 Apr 9;20(11):2637–46. doi: 10.1002/1878-0261.70234 (PMC13398454; doi:10.1002/1878-0261.70234)
Supplement: Supplementary file 1 [file MOL2-20-2637-s001.docx]

**SUPPORTING INFORMATION**

Table of contents

- **SUPPLEMENTARY TABLES & TABLE LEGENDS**
- **SUPPLEMENTARY REFERENCES**
- **SUPPLEMENTARY FIGURES & FIGURE LEGENDS**

**SUPPLEMENTARY TABLES AND TABLE LEGENDS**

**Supplementary Table 1.** **Therapeutically Applicable Research to Generate Effective Treatments (TARGET) initiative.** Details of the first patient subset that includes 265 T-cell acute lymphoblastic leukemia (T-ALL) pediatric cases from the TARGET initiative with available information regarding expression data of the tumor sample at diagnosis and minimal residual disease (MRD) levels after induction chemotherapy.

| **Feature** | **TARGET initiative (n=265)** |
| --- | --- |
| **Age at Diagnosis in Years Mean (range)** | 9.6 (1-29) |
| **Sex** | |
| Males | 202 (76.2%) |
| Females | 63 (23.8%) |
| **MRD** | |
| MRD < 0.01% (low) | 172 (64.9%) |
| 0.01% ≤ MRD ≤ 0.1% (medium) | 30 (11.3%) |
| MRD > 0.1% (high) | 63 (23.8%) |

**Supplementary Table 2.** **Gabriella Miller Kids First Pediatric Research Program.** Details of the second patient subset that includes 1070 additional T-cell acute lymphoblastic leukemia (T-ALL) pediatric cases from the Gabriella Miller Kids First Pediatric Research Program with available information regarding expression data of the tumor sample at diagnosis and minimal residual disease (MRD) levels after induction chemotherapy.

| **Feature** | **Gabriella Miller Kids First Pediatric Research Program (n=1070)** |
| --- | --- |
| **Age at Diagnosis in Years Mean (range)** | 9.5 (1-29) |
| **Sex** | |
| Males | 792 (74%) |
| Females | 285 (26.6%) |
| **MRD** | |
| MRD < 0.01% (low) | 620 (57.9%) |
| 0.01% ≤ MRD ≤ 0.1% (medium) | 68 (6.4%) |
| MRD > 0.1% (high) | 382 (35.7%) |

**Supplementary Table 3.** **Comparison between the transcriptional signature and the genomic subtypes established by Pölönen *et al*** [1] **in patients from Gabriella Miller Kids First Pediatric Research program.** The parameters analyzed were: proportion of high- minimal residual disease (MRD) patients, specificity and sensitivity. Only those subgroups significantly associated with a greater probability of experiencing an elevated MRD after induction chemotherapy were subsequently analyzed for specificity and sensitivity. Statistical significance was set at p<0.05.

| **Group** | **N** | **High MRD (%)** | **p-value** | **Enriched in** | **Specificity (%)** | **p-value** | **Sensitivity (%)** | **p-value** |
| --- | --- | --- | --- | --- | --- | --- | --- | --- |
| **Signature** | 301 | 78.1 | <0.0001 | High MRD | 89.4 | <0.0001 | 61.5 | <0.0001 |
| **Remainder** | 701 | 21.0 |  |  |  |  |  |  |
| **BCL11B** | 12 | 58.3 | 0.2297 | High MRD |  |  |  |  |
| **Non BCL11B** | 990 | 37.9 |  |  |  |  |  |  |
| **ETP-like** | 201 | 83.6 | <0.0001 | High MRD | 94.7 | <0.0001 | 44.0 | 0.992 |
| **Non ETP-like** | 801 | 26.7 |  |  |  |  |  |  |
| **HOXA9 TCR** | 17 | 17.6 | 0.128 | Low MRD |  |  |  |  |
| **Non HOXA9 TCR** | 985 | 38.5 |  |  |  |  |  |  |
| **KMT2A** | 22 | 81.8 | <0.0001 | High MRD | 99.4 | <0.0001 | 4.7 | >0.9999 |
| **Non-KMT2A** | 980 | 37.1 |  |  |  |  |  |  |
| **LMO2 γδ-like** | 10 | 100.0 | <0.0001 | High MRD | 100.0 | <0.0001 | 2.6 | >0.9999 |
| **Non LMO2 γδ-like** | 992 | 37.5 |  |  |  |  |  |  |
| **MLLT10** | 21 | 23.8 | 0.2555 | Low MRD |  |  |  |  |
| **Non MLLT10** | 981 | 38.4 |  |  |  |  |  |  |
| **NKX2-1** | 60 | 11.7 | <0.0001 | Low MRD | 91.5 |  | 1.8 |  |
| **Non NKX2-1** | 942 | 39.8 |  |  |  |  |  |  |
| **NKX2-5** | 6 | 83.3 | 0.0326 | High MRD | 99.8 | <0.0001 | 1.3 | >0.9999 |
| **Non NKX2-5** | 996 | 37.9 |  |  |  |  |  |  |
| **SPI1** | 10 | 20.0 | 0.3336 | Low MRD |  |  |  |  |
| **Non SPI1** | 992 | 38.3 |  |  |  |  |  |  |
| **STAG/LMO2** | 7 | 71.4 | 0.1128 | High MRD |  |  |  |  |
| **Non STAG/LMO2** | 995 | 37.9 |  |  |  |  |  |  |
| **TAL1 DP-like** | 231 | 11.7 | <0.0001 | Low MRD | 67.1 |  | 7.1 |  |
| **Non TAL1 DP-like** | 771 | 46.0 |  |  |  |  |  |  |
| **TAL1 αβ-like** | 164 | 34.8 | 0.3794 | Low MRD |  |  |  |  |
| **Non TAL1 αβ-like** | 838 | 38.8 |  |  |  |  |  |  |
| **TLX1** | 45 | 17.8 | 0.0042 | Low MRD | 94.0 |  | 2.1 |  |
| **Non TLX1** | 957 | 39.1 |  |  |  |  |  |  |
| **TLX3** | 151 | 33.8 | 0.2389 | Low MRD |  |  |  |  |
| **Non TLX3** | 851 | 38.9 |  |  |  |  |  |  |
| **TME-enriched** | 36 | 13.9 | 0.0024 | Low MRD | 95.0 |  | 1.3 |  |
| **Non TME-enriched** | 966 | 39.0 |  |  |  |  |  |  |
| **Other** | 9 | 44.4 | 0.7378 | Low MRD |  |  |  |  |
|  | 993 | 38.1 |  |  |  |  |  |  |

**SUPPLEMENTARY REFERENCES**

1	Pölönen P, Di Giacomo D, Seffernick AE, Elsayed A, Kimura S, Benini F, et al. The genomic basis of childhood T-lineage acute lymphoblastic leukaemia. *Nature*. 2024;**632**(8027):1082–1091.

**SUPPLEMENTARY FIGURES AND FIGURE LEGENDS**

**Supplementary Figure 1. Identification of candidate genes for the transcriptional signature.** (**A**) Volcano plot showing gene expression differences between high- and low- minimal residual disease (MRD) patients. Genes were considered differentially expressed based on an absolute log2 fold change greater than 1 and an adjusted p-value below 0.05. (**B**) Table listing the subset of 25 genes examined in the multiple correspondence analysis (MCA). All the selected genes were differentially expressed between high- and low-MRD patients (log_2_FC>|1| and p<0.05), participate in multiple biological processes that are relevant for T-cell homeostasis and have been previously implicated in cancer. (**C**) MCA for T-cell acute lymphoblastic leukemia (T-ALL) patients according to the differential expression of the gene subset previously specified in Supplementary Figure 1B. For each of the selected genes, patients were divided into quartiles according to their mRNA expression levels and subsequently classified as Q1 (lowest expression); Q2; Q3; or Q4 (highest expression). Only those patients with MRD values classified as either high or low were considered for analysis. (**D**) Individual component scores for clusters 1 and 2 obtained in the MCA of Supplementary Figure 1C. Data were analyzed using Mann Whitney test and statistical significance was set at p<0.05. (**E**) Boxplot comparing MRD levels after induction chemotherapy between patients from cluster 1 and cluster 2. Data were analyzed using Mann Whitney test and statistical significance was set at p<0.05.

**Supplementary Figure 2. Transcriptional signature in T-cell acute lymphoblastic leukemia (T-ALL) patients from Therapeutically Applicable Research to Generate Effective Treatments (TARGET) initiative.** (**A**) Boxplot analyzing mRNA expression levels for *BCL2*, *HSH2D*, *LAT2*, *MAST4*, *METRN* and *PITPNM2* genes in T-ALL patients with high or low minimal residual disease (MRD). Data were analyzed using Mann Whitney test and statistical significance was set at p<0.05. (**B**) Association analysis between paired gene-expression states in high-MRD patients. Relationships between *HSH2D* and *LAT2* upregulation (left), *BCL2* and *MAST4* upregulation (middle), and *METRN* and *PITPNM2* downregulation (right) are shown. For each gene pair, the proportion of patients exhibiting concordant expression states (simultaneous upregulation or downregulation of both genes) was compared with the proportion of patients exhibiting discordant expression states (upregulation of one gene and downregulation of the other). Statistical associations between gene-expression combinations were evaluated using the chi-square test and corrected using the Benjamini-Hochberg multiplicity test. The data are represented as percentages with 95% confidence intervals (Wilson method). Statistical significance was set at p<0.05.

**Supplementary Figure 3. Transcriptional signature in T-cell acute lymphoblastic leukemia (T-ALL) patients from Gabriella Miller Kids First Pediatric Research Program.** (**A**) Individual component scores for clusters 1 and 2 obtained in the multiple correspondence analysis (MCA) of Figure 2D. Data were analyzed using Mann Whitney test and statistical significance was set at p<0.05. (**B**) Boxplot analyzing mRNA expression levels for *BCL2*, *HSH2D*, *LAT2*, *MAST4*, *METRN* and *PITPNM2* genes in T-ALL patients with high or low minimal residual disease (MRD). Data were analyzed using Mann Whitney test and statistical significance was set at p<0.05. (**C**) Association analysis between paired gene-expression states in high-MRD patients. Relationships between *HSH2D* and *LAT2* upregulation (left), *BCL2* and *MAST4* upregulation (middle), and *METRN* and *PITPNM2* downregulation (right) are shown. For each gene pair, the proportion of patients exhibiting concordant expression states (simultaneous upregulation or downregulation of both genes) was compared with the proportion of patients exhibiting discordant expression states (upregulation of one gene and downregulation of the other). Statistical associations between gene-expression combinations were evaluated using the chi-square test and corrected using the Benjamini-Hochberg multiplicity test. The data are represented as percentages with 95% confidence intervals (Wilson method). Statistical significance was set at p<0.05.

**Supplementary Figure 4. The differential expression of *HSH2D*&*LAT2*/*BCL2*&*MAST4* /*METRN*&*PITPNM2* is associated with high- minimal residual disease (MRD) genomic subtypes.** (**A**) Proportion of patients with genomic subtypes associated with high MRD, low MRD or none in the transcriptional signature group (left) and the remainder group (right). MRD low: <0.01%; MRD medium: 0.01%-0.1%; MRD high: >0.1%. The transcriptional signature involves those cases with simultaneous upregulation (Q4) of either *HSH2D* and *METRN* or *BCL2* and *MAST4* or simultaneous downregulation (Q1) of *METRN* and *PITPNM2*. According to Supplementary Table 3, the genomic subtypes associated with high MRD (red) are ETP-like, KMT2A, LMO2 γδ-like and NKX2-5 while the genomic subtypes associated with low MRD (blue) are NKX2-1, TAL1 DP-like, TLX1 and TME-enriched. The remaining genomic subtypes were not significantly enriched in high/low-MRD patients and were labelled as “Other genomic subtypes” (white). (**B**) The proportions of patients from high- and low-MRD genomic subtypes were compared between the transcriptional signature group and the remainder group. The graph shows the percentage of patients in each group with 95% confidence intervals (Wilson method). Statistical significance was set at p<0.05.
